# OSABSS: An authentic examination for assessing basic surgical skills in surgical residents

**DOI:** 10.1016/j.sopen.2024.04.008

**Published:** 2024-05-13

**Authors:** Leila Sadati, Fatemeh Edalattalab, Niloofar Hajati, Sahar Karami, Ali Baradaran Bagheri, Mohammad Hadi Bahri, Rana Abjar

**Affiliations:** a Department of Operating Room, School of Paramedical Sciences, Alborz University of Medical Sciences, Karaj, Iran; b School of Paramedical Sciences, Alborz University of Medical Sciences, Karaj, Iran; c Medical education department, School of medicine, Tehran University of Medical Sciences, Tehran, Iran; d Department of Neurosurgery, School of Medicine, Shahid Madani Hospital, Alborz University of Medical Sciences, Karaj, Iran; e Department of Surgery, Shahid Madani Hospital, School of Medicine, Alborz University of Medical Sciences, Karaj, Iran

**Keywords:** Residency, Surgery, Competency, Assessment, Validity, Reliability

## Abstract

**Objectives:**

This study aimed to develop and validate the OSABSS (Objective Structured Assessment of Basic Surgical Skills), a modified Objective Structured Clinical Examination (OSCE), to assess basic surgical skills in residents.

**Design:**

A developmental study conducted in two phases. Basic skills were identified through literature review and gap analysis. The OSABSS was then designed as a modified OSCE.

**Setting:**

This study took place at Alborz University of Medical Sciences in Iran.

**Interventions:**

The OSABSS was created using Harden's OSCE (Objective Structured Clinical Examination) methodology. Scenarios, checklists, and station configurations were developed through expert panels. The exam was piloted and implemented with residents as participants and faculty as evaluators.

**Participants:**

32 surgical residents in gynecology, general surgery, orthopedics, and neurosurgery participated. 22 faculty members were evaluators.

**Primary and secondary outcome measures:**

The primary outcome was OSABSS exam scores. Secondary outcomes were written exam scores, and national residency entrance ranks.

**Main results:**

The mean OSABSS score was 16.59 ± 0.19 across all stations. Criterion validity was demonstrated through correlations between OSABSS scores, written scores and entrance ranks. Reliability was high, with a Cronbach's alpha of 0.87. No significant inter-rater score differences were found.

**Conclusions:**

The rigorous OSABSS development process produced an exam demonstrating strong validity and reliability for assessing basic surgical skills. The comprehensive station variety evaluates diverse technical and non-technical competencies. Further research should expand participant samples across surgical disciplines.

## Introduction

While the medical education literature does not offer a comprehensive, standardized, and universally accepted definition of surgical competence, there is a consensus that certain technical and non-technical skills are integral for a surgeon to execute safe, independent surgeries [1,2]. Definitions such as the CanMEDS description of an expert surgeon, along with recommendations from the Royal College of Physicians and Surgeons of Canada (2015) [3], the Royal Australasian College of Surgeons, and the Royal College of Surgeons of England, emphasize technical skills necessary for safe surgery. These include tissue access, hemostasis establishment, and wound care, in addition to non-technical skills like management, teamwork, and communication [4,5]. However, the overlapping of these competencies and the lack of clarity on how to convert them into measurable skills and abilities are also serious challenges in medical education [6].

On the other way, as per the model proposed by Sadati et al., basic surgical skills form a fundamental component of surgical competence [7]. These skills encompass wearing surgical gowns and gloves, hand scrubbing, using surgical instruments and equipment, suturing, knot tying, prep, and draping, among others [8]. Only a few studies, such as those by Vedula, Gonzalez, and Oram, have highlighted these skills, particularly suturing, knot-tying, and the donning of surgical gowns and gloves [9,10]. Hence, these essential skills are often overlooked in training programs for surgical residents and in their assessments, where the focus is largely on evaluating technical skills like skin incision, T-tube insertion, abdominal wall closure, hand-sewn intestinal anastomosis, stapled intestinal anastomosis, hemostasis, pyloroplasty, and tracheostomy in a real situation [[11], [12], [13], [14]]. Common assessment criteria include the duration of each surgical stage, the number of movements executed, hemostasis control, tissue access, and the instruments employed in performing specific surgeries such as appendectomy and cholecystectomy [15]. As a result, it is necessary to create an appropriate assessment to measure basic surgical skills.

A review of medical education texts reveals that an Objective Structured Clinical Examination (OSCE) can effectively assess some of these basic skills, such as suturing and surgical dressing [[16], [17], [18]]. But, considering the educational objectives, it is essential to create a variety of stations, checklists, and scenarios. Hence, medical schools have progressively endeavored to enhance the quality of this examination by devising different scenarios and stations tailored to students' educational levels, with appropriate scoring rubrics and trained evaluators [[19], [20], [21], [22]].

Given the importance of teaching and evaluating both technical and non-technical skills in surgical residents, and considering that acceptance into specialty programs primarily evaluates specific cognitive abilities and specialized knowledge while often overlooking basic skills, there is a pressing need for a more holistic assessment approach. To address this gap, the authors first designed a specialized boot camp to teach basic surgical skills (results will be reported separately). They then developed the Objective Structured Assessment of Basic Surgical Skills (OSABSS) - an innovative, modified OSCE to evaluate these skills. The OSABSS serves as a comprehensive, reliable and valid measure of fundamental surgical competence that ensures surgeons are adequately equipped with essential technical and non-technical abilities. This manuscript elaborates on the rigorous development and demonstrates preliminary efficacy of the OSABSS as an authentic competency examination tailored for assessing basic surgical skills in surgical residents.

## Material and methods

### Multi-phase OSABSS development methodology

This iterative, robust development process systematically built an innovative surgical resident assessment from the ground up over six interrelated but methodical phases.

Phase 1- Building a Comprehensive Foundation of Skills: The first phase focused on identifying the necessary abilities and competencies. This was done through a thorough review of medical education literature, high-stakes surgical exams, and accreditation documents. This phase put together the range of benchmark technical skills, such as sterile gloving procedures, knot tying and suturing. It also included critical non-technical skills like ethical decision-making, situation awareness, communication, and professionalism. Additionally, it outlined important skills that current evaluations and training often miss, even though specialty boards emphasize integrating these skills before independent clinical practice.

Phase 2– Developing a Consensus Blueprint: Building on the foundation of surgical skills identified in Phase 1, a panel of six specialized surgeons from fields including orthopedics, neurosurgery, gynecology, and general surgery worked together iteratively to decide which competencies should be included. This was done to address gaps identified in current residency training assessments. Using a targeted needs assessment methodology, they systematically evaluated the aggregated skills list. Over multiple consensus meetings, they debated and refined the evolving list of skills. By applying insights from their diverse surgical experience with real-world residency training challenges, additional essential skills were added. Finally, the group reached consensus on a final set of 28 foundational baseline skills to assess. This formal consensus blueprinting process by the multi-disciplinary panel further reinforced basing the OSABSS exam components on the most relevant, agreed-upon set of surgical skill competencies.

Phase 3- Mapping Skills to Exam Stations: With the foundational skills established through the consensus blueprinting process, measurement experts proceeded to map them to exam stations for the in-development OSABSS exam. Applying best practices in structured competency assessment design, they developed robust specifications for 15 formative assessment stations. These stations covered the most multifaceted yet interrelated facets of the required surgical competencies. Station topics included fundamental sterile techniques like gowning/gloving, essential abilities such as suturing/knot-tying, and the integration of professional non-technical skills. These were woven across stations dealing with ethical decision-making scenarios, situation awareness, and targeted communication sequences. Table 1 outlines the measurement mapping between the expert-agreed upon focus skills/skillsets and the corresponding OSABSS exam stations. This enables consistent, standardized assessment of the skills.

**Table 1:** List of examination stations along with the designated space for each station.

Dedicated space for the station	Station number	Station titles
A	1.	Hand rub
A	2.	Hand scrub
B	3.	Donning gowns
B	4.	Donning gloves
C	5.	Doffing gowns
C	6.	Doffing gloves
D	7.	Surgical prep
D	8.	Surgical drape
E	9.	Rest
F	10.	Knot tying
F	11.	Suturing
G	12.	surgical table setup
H	13.	Surgical dressing
I	14.	Surgical positioning
J	15.	Problem solving
K	16.	Decision making

**Fig. 1:**
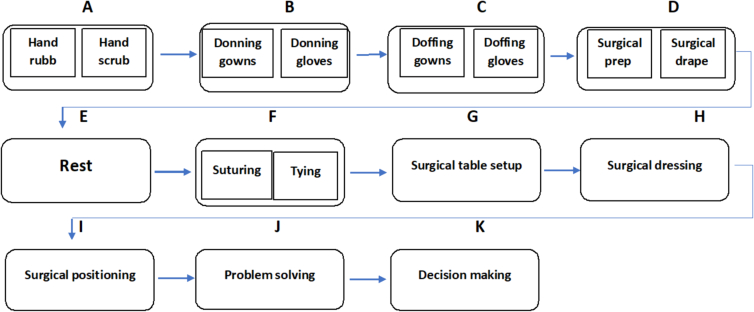
The movement path of surgical residents during the examination process at the stations.

Phase 4– Developing Customized Assessment Scenarios: With the focus competencies systematically mapped to the associated OSABSS exam stations, medical education experts collaborated with experienced surgeons. Together, they developed 100 customized scenarios designed to reliably and validly evaluate the outlined skills within each station's context. Additionally, teams crafted intricate corresponding checklists to gauge gradations of competency attainment for every focus skill. This enabled robust quantitative evaluation and detailed qualitative feedback. For example, the “Donning Gloves” station scenario contained specifics allowing evaluators to consistently rate related techniques, sterile adherence, and sequence completion. Some sequential skill stations, like proper gowning/gloving techniques, were strategically grouped together in adjoining spaces. This maximized the use of the valuable expert evaluator resources, with two simultaneous trained raters evaluating each resident at combined stations. Fig. 1 visually depicts the precise sequence and flow of rotating resident groups across the OSABSS stations.

Phase 5– Rigorous Pilot Testing and Refinement: After extensively blueprinting OSABSS competencies, mapping skills to stations, developing scenarios and checklists, and configuring space requirements, specialized rater training was conducted. This standardized understanding of scenario details, the specific skill components needing assessment, and nuances across rating scales. Following training, the extensive 15-station OSABSS exam underwent a rigorous pilot study with non-resident volunteers across surgical disciplines. This critical quality assurance benchmark enabled identifying and correcting any inconsistencies in scenario configurations before full deployment, further refining flow and enhancing the clarity of task instructions. Technically trained actors also ensured scenarios elicited appropriate cues to reliably evaluate each competency. Analysis from pilot testing provided pivotal feedback to streamline continuity and resolve remaining ambiguities prior to the full launch.

Phase 6– Psychometric Benchmarking for High-Stakes Decisions: After iterative refinements enhanced standardized delivery, 32 surgical residents across specialties completed the full 15-station OSABSS competency exam. Two trained faculty evaluators used precisely developed scenarios and tailored checklists to assess each resident's performance, evaluating the outlined skills both quantitatively and qualitatively. The checklists utilized behaviorally-anchored rating scales, ranging from 1 to 5 or 1 to 20 points, tailored to each skill set. Robust quantitative psychometric analysis enabled precise evaluation of the tools' measurement properties based on response patterns. This supports the interpretation for high-stakes decisions on readiness for independent surgical practice.

### Statistical analyses

We performed a robust quantitative analysis using SPSS Version 22 software to evaluate the measurement properties upon full administration to participants. Psychometric benchmarking empirically analyzed result patterns to provide evidence supporting the interpretation of scores for high-stakes evaluation of surgical readiness.

*Reliability Appraisal*: We performed several types of analysis to evaluate the reliability of the OSABSS exam scores. First, we looked at the internal reliability by examining the correlation between trainees' total test scores and their scores on individual exam stations. High correlations here would indicate that all the stations are consistently measuring the same underlying surgical skills and knowledge. Second, we had two trained evaluators simultaneously rate residents' performance during some stations to gauge inter-rater consistency. A high correlation between the two evaluators' scores would show consensus in how the exam is being scored across observations.

Additionally, we calculated Cronbach's alpha for the stations collectively to quantify the internal consistency of the whole exam. Cronbach's alpha measures how closely related sets of station scores are to each other, factoring out random errors in measurement. Resulting alpha values range from 0 to 1 - scores above 0.7 indicate good internal reliability. We used established benchmarks to interpret whether our exam's alpha level demonstrated adequate reliability for high-stakes decisions about residents' surgical readiness.

*Validity Assessment*: We used several quantitative methods to evaluate the validity of the OSABSS exam scores from multiple perspectives. First, to assess face and content validity, we had an expert panel review the exam stations, checklists, and measurement mappings. Their review confirmed that the scenarios and evaluations make sense for testing the key surgical skills originally identified as targets when developing the exam blueprint. Second, for construct validity, we analyzed if trainees' competence levels (scores) on individual stations correlated with their total test performance. Finding that station scores correlated with overall scores would provide evidence that the exam is measuring connected underlying constructs. Third, for criterion validity, we compared OSABSS scores to performance on two existing exams. Doing well on the competitive national residency entrance exam enables matching into more prestigious residency programs. This likely reflects stronger baseline abilities that reasonably relate to later surgical competence. Also, an exam directly testing retained knowledge from earlier training links to applied skills acquisition.

## Results

The study included 32 assistant participants, comprising 40.6 % (13 individuals) females and 59.4 % (19 individuals) males. The average age of the participants was 32.15 ± 3.59 years, ranging from 26 to 40 years, and their professional experience averaged 1.21 ± 0.42 years, within the range of 1–2 years. Of the total, 37.5 %.

Upon analyzing the residents' scores in the examination, the mean score across all stations was determined to be 16.59 ± 0.19. The lowest score was achieved by the gynecology group, with a score of 16.44 ± 0.16, while the highest was scored by the neurosurgery group, at 16.89 ± 0.14.

In assessing the examination's criterion validity, a correlation was established between the OSABSS score, the written examination scores, and the national residency entrance rank. A direct correlation was found among these variables, which validated the examination's criterion validity (*P* < 0.001) (Table 2).

**Table 2:** Correlation between the OSABSS score and the scores of the written examination and the national residency entrance rank.

Group	B	SE	Beta	t	P
Constant	15.72	0.22		71.08	<0.001
Written score	0.006	0.01	0.47	4.26	<0.001
*R* = 0.47		R^2^ = 0.22		ADJ.R^2^ = 0.214	
Constant	17.08	0.05		343.80	<0.001
Rank of assistance	−0.15	0.01	−0.75	−9.08	<0.001
*R* = 0.75		R^2^ = 0.57		ADJ.R^2^ = 0.564	

The independent variable of written examination scores explained 21.4 % of the variance in the mean total scores, while the independent variable of the resident's rank accounted for 56.4 % of the variance. The practical score increased by 0.47 units with every one-unit increase in the written examination score, and the practical score increased by 0.75 units with every one-unit decrease in the resident's rank (indicating a better rank).

**Chart 1:**
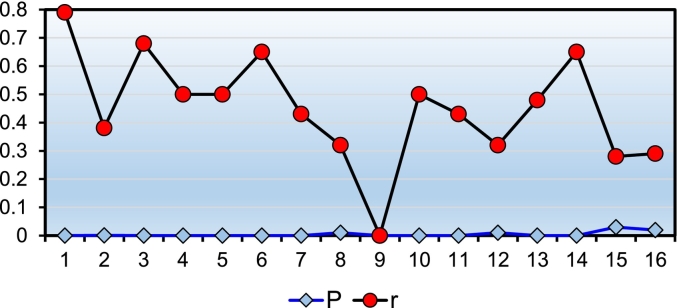
Correlation between the average total score and different stations.

Spearman correlation coefficient analysis for internal consistency revealed a significant positive correlation between the mean total score and the scores of each station, suggesting that an increase in the scores of each station led to an increase in the mean total score, as shown in Chart 1. The correlation strength varied among stations, with station 1 showing a strong correlation and others ranging from weak (stations 15, 16) to moderate (other stations). Despite the small differences in scores among stations, the findings indicate a stronger correlation between technical skills and the total score.

**Chart 2:**
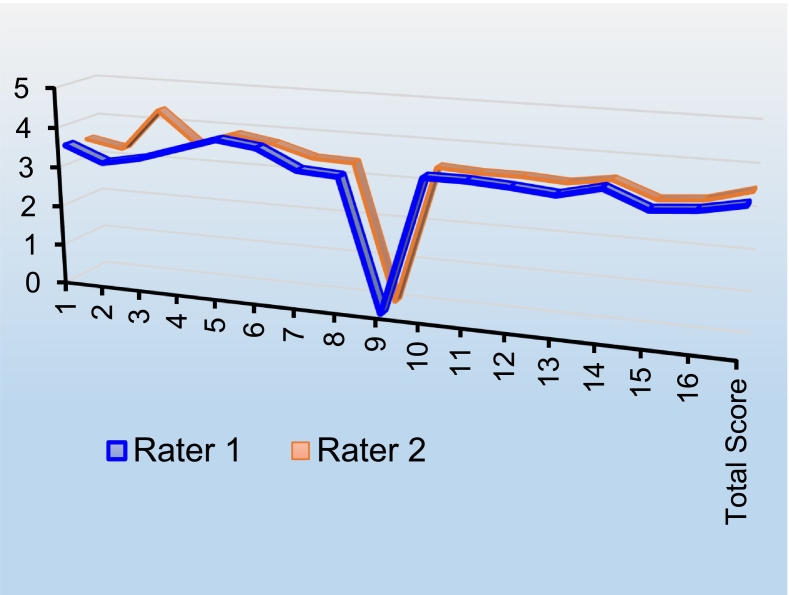
The degree of evaluators' agreement in global scoring.

The Cronbach's alpha coefficient was calculated as 0.87, suggesting a high reliability level for the exam. Furthermore, a comparison using the *t*-test and chi-square test between all residents' scores, according to the evaluators, revealed no significant differences at any of the stations (*P* > 0.05), suggesting consistent evaluation. The Spearman correlation coefficient indicated a highly significant positive correlation between the first and second evaluators' global scores, as depicted in Chart 2.

## Discussion

This study aimed to develop and validate a competency exam known as the OSABSS. This exam is a modified version of the OSCE designed to assess the basic surgical skills of surgical residents. The results of the study demonstrated that the OSABSS possesses the necessary qualifications, including validity and reliability, making it suitable for the assessment of basic surgical skills in surgical residents. The importance of developing and implementing OSCEs in residency programs has been highlighted in various studies. For instance, Martin developed the OSTAS tool for assessing specific technical skills in surgery, including actions such as skin incision, T-tube insertion, abdominal wall closure, manual and stapled anastomosis of the intestine, bleeding control, pyloroplasty, and tracheostomy [14]. The Ottawa Surgical Competency Operating Room Evaluation (O-SCORE) is another surgical evaluation tool designed to assess technical competence in surgical trainees using behavioral anchors. This tool consists of 9 items [12]. In line with this, Kassam A and colleagues have suggested that implementing OSCEs in residency programs can aid in evaluating professional competencies [20]. Similar views are expressed by Melcher P et al., emphasizing the importance and implementation requirements of the OSCE exam in orthopedic residents [23]. In another study, Phillips D and colleagues developed an OSCE exam aligned with the milestones of the orthopedic education course and the six competencies approved by the ACGME [24].

When designing objective structured clinical exams, it is of utmost importance to pay attention to the quality of exam administration. In this regard, Warner DO and colleagues emphasize the importance of the OSCE in evaluating the clinical performance of anesthesia trainees and recommend that the exam design should include initial conceptualization, OSCE structure, scenario development process, standardized patient preparation, evaluator training, and scoring and evaluation procedures. They also stress the need for meticulous attention to the quality of exam administration [21]. In a similar vein, Melcher P, Roth A, and colleagues highlight similar issues, including evaluator training, standardized patients, appropriate scenario design, and checklists in evaluating exam quality [23]. The AMEE Guideline 81 provides detailed guidance on the process of scenario development, station preparation, evaluator training, and scoring and evaluation procedures. In the development process of this exam, we adhered to standard guidelines and incorporated insights from other studies to ensure its quality [22,25].

The number of stations considered in different studies for educational purposes varies between 6 and 16, with each station allocated a duration of five or 10 min. Lee et al. considered six ten-minute stations for their exam, which is similar in terms of time allocation to the present study [26]. However, in the present study, a 15-station exam was designed based on the course blueprints and educational objectives. Khan et al. suggest that increasing the number of stations leads to increased validity and reliability of the exam, which is one of the strengths of the present exam [22]. Joong Hiong Sim et al. designed an ASCI exam with 16 stations and one rest station, each with a duration of 5 min, to evaluate the performance of medical students in different stations [27]. Swanson also emphasizes that increasing the number of stations can improve the reliability of exam [28]. The variety and high number of technical and non-technical skills in the field of the operating room are among the strengths of this study, which distinguish it from previous studies in terms of the number of stations and make it unique.

According to medical education pioneers, reliability and validity are key elements in the quality of structured clinical exams. Based on the results of the present study, the intra-group correlation coefficient was between 0.4 and 0.8 for more than half of the technical skill stations and about 3 % for the two non-technical skill stations 15 and 16. This indicates a medium to strong correlation in technical skills and a weak correlation with non-technical skill stations, thereby confirming the internal consistency of the exam. In the criterion validity analysis of the examination, the correlation between the OSABSS score and, the scores of the written examination and the national residency entrance rank was found to be significant with *p* < 0.001. Specifically, 21.4 % of the variance in the mean overall scores was predicted by the independent variable of written exam scores, and 56.4 % of the variance in the mean overall scores was predicted by the independent variable of individual rank. An increase of one unit in written exam scores was associated with a 0.47-unit increase in practical exam scores, and a decrease of one unit in individual rank (achieving a better rank) was associated with a 0.75-unit increase in practical exam scores. Several studies, including those by Lee et al., Sahebalzamani et al., Roshanzadeh et al., Ghiasi, Biranvand et al., and Dehnoulian et al., have referred to two indices when evaluating the reliability of OSCE: Cronbach's alpha and the percentage of agreement among evaluators at selected or all stations [26,[29], [30], [31], [32], [33]]. In the present study, these indices were used as the evaluation criteria for the reliability of the exam. The Cronbach's alpha calculated in this study was 87 %, and the inter-rater agreement coefficient for the scores given at each station was over 90 %, indicating the reliability of the designed exam.

In addition to the high number of stations, the strengths of this exam include the design of diverse stations, the simultaneous presence of two evaluators at each station, and consideration of a variety of basic technical skills in different stations. These factors provide a more reliable picture of a learner's overall competence. Furthermore, as learners moved through all stations, they were assessed by different examiners, and the collection of multiple independent observations reduced individual biases and prejudices. This is consistent with the findings of Joong Hiong Sim et al. [27]. Also, in this study, the researchers also considered a global score for the learner's competence at each station, in addition to the score assigned for each checklist at each station, which aligns with the study by Kassam et al. [20]. The actions taken in administering the exam are consistent with previous studies and represent a standard path in developing structured clinical exams, indicating acceptable quality in implementing this exam.

## Strengths and limitations

A major strength of this study was the rigorous, comprehensive development process for the OSABSS exam, which incorporated literature review, expert input, and pilot testing, resulting in a robust assessment tool evaluating diverse basic surgical skills across 15 stations. Additional strengths include the demonstration of strong validity and inter-rater reliability through multiple analytical methods. However, there are some limitations, including the small sample size of residents from a single academic medical center, which restricts generalizability and applicability to other settings and surgical disciplines. Other limitations are the potential variability in residents' prior experiences impacting their exam scores, as well as the intensive resource requirements, which may affect the feasibility for some programs. Overall, the OSABSS shows significant potential as a valuable assessment tool for basic surgical skills training, but further research should aim to expand the sampling across institutions and specialties in order to increase generalizability.

## Conclusion

This study successfully designed, developed, and validated the OSABSS, a comprehensive examination tailored for assessing basic surgical skills among surgical residents. The OSABSS stands out for its extensive range of stations, enabling a thorough assessment of both technical and non-technical skills and thereby enhancing its reliability and holistic evaluation of learners' competencies. The positive correlation between OSABSS scores and other measures of academic achievement, such as written examination scores and national residency entrance ranks, underscores its predictive validity. The reliability of the OSABSS is further supported by strong inter-rater agreement and internal consistency measures.

While acknowledging the study's limitations, notably the small and homogenous sample size, this research lays a solid foundation for the OSABSS as a valuable tool in surgical education. It underscores the need for further validation studies across more diverse and larger populations to solidify its applicability in different contexts and surgical disciplines. The OSABSS has the potential to significantly enhance surgical training programs by providing a reliable, valid, and comprehensive method for assessing essential surgical competencies. Future efforts should focus on expanding the use and evaluation of the OSABSS to continue improving surgical education and, ultimately, patient care outcomes.

## Funding sources

This project was partially funded through institutional funding (10.13039/501100012411Alborz University of Medical Sciences).

## Ethical approval statement

This study was approved by the Alborz University of Medical Sciences Research Ethics Committee, under approval number IR.ABZUMS.REC.1400.278. Informed consent was obtained from all individual participants included in the study.

## CRediT authorship contribution statement

**Leila Sadati:** Conceptualization, Data curation, Formal analysis, Investigation, Methodology, Project administration, Resources, Software, Supervision, Validation, Visualization, Writing – original draft, Writing – review & editing. **Fatemeh Edalattalab:** Conceptualization, Data curation, Investigation, Methodology, Resources, Software, Validation, Visualization, Writing – review & editing. **Niloofar Hajati:** Conceptualization, Data curation, Investigation, Methodology, Resources, Software, Validation, Visualization, Writing – review & editing. **Sahar Karami:** Conceptualization, Data curation, Investigation, Methodology, Resources, Software, Validation, Visualization, Writing – review & editing. **Ali Baradaran Bagheri:** Conceptualization, Formal analysis, Investigation, Methodology, Resources, Software, Validation, Visualization, Writing – review & editing. **Mohammad Hadi Bahri:** Conceptualization, Formal analysis, Investigation, Methodology, Resources, Software, Validation, Visualization, Writing – review & editing. **Rana Abjar:** Conceptualization, Data curation, Formal analysis, Investigation, Methodology, Project administration, Resources, Software, Supervision, Validation, Visualization, Writing – original draft, Writing – review & editing.

## Declaration of competing interest

The authors declare that they have no conflict of interest.

## Data Availability

The datasets generated and/or analyzed during the current study are not publicly available due [REASON WHY DATA ARE NOT PUBLIC] but are available from the corresponding author on reasonable request.
